# Association of resting heart rate and its change with incident cardiovascular events in the middle-aged and older Chinese

**DOI:** 10.1038/s41598-019-43045-5

**Published:** 2019-04-25

**Authors:** Jing Tian, Yu Yuan, Miaoyan Shen, Xiaomin Zhang, Meian He, Huan Guo, Handong Yang, Tangchun Wu

**Affiliations:** 1 Key Laboratory of Environment and Health, Ministry of Education and State Key Laboratory of Environmental Health (Incubating), School of Public Health, Tongji Medical College, Huazhong University of Science and Technology, Wuhan, Hubei China; 2 Dongfeng Central Hospital, Dongfeng Motor Corporation and Hubei University of Medicine, Shiyan, Hubei China

**Keywords:** Cardiovascular diseases, Risk factors

## Abstract

Whether heart rate change is associated with cardiovascular disease (CVD) in the general population is unclear. We conducted a prospective cohort study to assess the association of resting heart rate and its change with incident CVD in the middle-aged and older Chinese. Resting heart rate was measured during the baseline survey (September 2008 to June 2010) and the resurvey (2013). Incident CVD was followed up until December 31, 2016. Finally, a total of 20,828 participants were included in the analyses of baseline heart rate and 9132 participants were included in the analyses of heart rate change. The associations of baseline heart rate and heart rate change with incident CVD were assessed with multivariable Cox proportional hazards models. Compared with moderate baseline heart rate (65 to 80 bpm), low baseline heart rate (<65 bpm) was associated with higher risk of CVD (HR, 1.19; 95% CI, 1.07–1.32). Compared with stable heart rate (−5 to 15 bpm) in the moderate baseline heart rate group (65 to 80 bpm), an increase of heart rate (>15 bpm) in high baseline heart rate group (>80 bpm) (HR, 1.67; 95% CI, 1.02–2.71) or a decrease of heart rate (<−5 bpm) in low baseline heart rate group (<65 bpm) (HR, 2.48; 95% CI, 1.27–4.82) was associated with higher risk of CVD. In conclusion, low resting heart rate is associated with higher risk of CVD. Both continuous increase in high baseline heart rate and decrease in low baseline heart rate are associated with higher risk of CVD.

## Introduction

As a low-tech indicator of cardiac function, resting heart rate reflects the balance of sympathetic and parasympathetic activity^1^. Heart rate is an important determinant of cardiac rhythm^2^ as elevated heart rate has been shown to be associated with cardiovascular disease (CVD) incidence^3,4^ and mortality^3^ in the general population. The Framingham study included 4058 participants without known cardiovascular disease, and they found that per 1-standard deviation (SD) (11 bpm) elevated heart rate was associated with higher risk of CVD and mortality over 20 years follow-up^3^. An elevated heart rate has also been associated with poorer prognosis in patients with hypertension^5^ or acute coronary syndrome (ACS)^6^. Paul *et al*. found elevated heart rate was associated with higher risk of all-cause mortality among 4065 hypertensive patients^5^. These studies only measured resting heart rate once, while resting heart rate experiences fluctuation over time.

Resting heart rate is modifiable under the influence of many factors, including age, gender, race, genetic factors, blood pressure, blood lipid, blood glucose, physical activity, smoking and alcohol^1^. In studies of the general population, an increase in heart rate from preceding visit was associated with higher risk of mortality^7,8^. The ARIC study included 15,680 participants, and suggested that every 5-bpm increases in heart rate was associated with 12% higher risk of all-cause mortality^7^. In studies of patients with hypertension or heart failure (HF), an increase in heart rate was associated with increased case fatality^5,9^. The CHARM study included 7599 patients with HF, and found that every 5-bpm increases in heart rate was associated with 9% higher risk of all-cause mortality^9^. Furthermore, heart rate reduction was associated with improved prognosis in patients with coronary heart disease (CHD) or HF^10,11^. Modifiable resting heart rate is a low-tech predictor of adverse outcomes in patient groups^5,9–11^. However, it is lack of study investigating the association of heart rate change with CVD in the general population.

The objective of this study was to prospectively examine the associations of resting heart rate and its change with incident CVD and its subtypes (including CVD, CHD, ACS, ischemic stroke and hemorrhagic stroke) in a large population-based cohort of the middle-aged and older Chinese.

## Results

### Basic characteristics by baseline heart rate and change of heart rate

Of 20,828 participants who were included in the analyses of baseline heart rate, the mean (SD) age was 61 (8) years and 8805 (42.3%) were men. The median value of baseline heart rate was 73 bpm (IQR, 69 to 80 bpm). The distribution of baseline heart rate was summarized in Suppl. Fig. 2a. Participants with high baseline heart rate were more likely to be younger, physically inactive, with higher prevalence of hypertension, diabetes, hyperlipidemia and CVD family history (Table 1). Correlation analyses showed that heart rate was negatively associated with age, body mass index, and positively associated with systolic blood pressure, diastolic blood pressure, fasting blood glucose and triglyceride. Men had lower mean heart rate than women. Active people had lower mean heart rate than those who do not exercise (Suppl. Tables 4 and 5).

**Table 1:** Basic characteristics of participants by baseline heart rate and change of heart rate.

Variables	Baseline heart rate				Change in heart rate			
	<65 bpm	65 to 80 bpm	>80 bpm	*P* Value^a^	<−5 bpm	−5 to 15 bpm	>15 bpm	*P* Value^a^
No.	2211	14472	4145		1722	5984	1426	
Age, mean (SD), years	62.0 (7.9)	60.8 (7.8)	60.2 (8.2)	<0.001	61.6 (7.2)	61.0 (7.2)	61.5 (7.6)	0.39
Men, No. (%)	1127 (51.0)	5903 (40.8)	1775 (42.8)	<0.001	829 (48.1)	2443 (40.8)	566 (39.7)	<0.001
BMI, mean (SD), kg/m^2^	23.8 (3.1)	23.9 (3.2)	23.5 (3.3)	<0.001	23.9 (3.1)	23.9 (3.2)	23.7 (3.3)	0.14
Smoking status, No. (%)^b^								
Current smoker	518 (23.4)	2667 (18.4)	785 (18.9)	0.01	384 (22.3)	1085 (18.1)	236 (16.5)	<0.001
Former smoker	218 (9.9)	1225 (8.5)	389 (9.4)		163 (9.5)	508 (8.5)	115 (8.1)	
Never smoker	1471 (66.5)	10493 (72.5)	2961 (71.4)		1169 (67.9)	4369 (73.0)	1069 (75.0)	
Alcohol intake status, No. (%)^c^								
Current drinker	602 (27.2)	3449 (23.8)	1085 (26.2)	0.91	450 (26.1)	1392 (23.3)	280 (19.6)	<0.001
Former drinker	80 (3.6)	574 (4.0)	158 (3.8)		66 (3.8)	222 (3.7)	50 (3.5)	
Never drinker	1526 (69.0)	10428 (72.1)	2895 (69.8)		1204 (69.9)	4367 (73.0)	1096 (76.9)	
Education level, No. (%)								
Primary school or below	485 (21.9)	3371 (23.3)	790 (19.1)	0.08	516 (30.0)	1658 (27.7)	415 (29.1)	0.26
Middle school	767 (34.7)	5335 (36.9)	1533 (37.0)		635 (36.9)	2234 (37.3)	517 (36.3)	
High school or beyond	939 (42.5)	5655 (39.1)	1798 (43.4)		560 (32.5)	2041 (34.1)	482 (33.8)	
Physical activity (yes), No. (%)^d^	1604 (72.5)	10162 (70.2)	2818 (68.0)	<0.001	1196 (69.5)	4253 (71.1)	1021 (71.6)	0.17
CVD family history, No. (%)^e^	214 (9.7)	1564 (10.8)	504 (12.2)	0.001	148 (8.6)	521 (8.7)	121 (8.5)	0.93
Hypertension, No. (%)^f^	604 (27.3)	4863 (33.6)	1888 (45.5)	<0.001	591 (34.3)	1852 (30.9)	464 (32.5)	0.21
Hyperlipidemia, No. (%)^g^	727 (32.9)	4960 (34.3)	1559 (37.6)	<0.001	637 (37.0)	2107 (35.2)	557 (39.1)	0.32
Diabetes, No. (%)^h^	236 (10.7)	1668 (11.5)	687 (16.6)	<0.001	191 (11.1)	709 (11.8)	249 (17.5)	<0.001
Baseline heart rate, mean (SD), bpm	60.5 (2.6)	72.8 (4.7)	89.2 (7.9)	<0.001	79.6 (10.2)	71.8 (7.8)	69.2 (7.4)	<0.001

Abbreviations: BMI, body mass index; CVD, cardiovascular disease.

^a^For linear trend, regression analyses were used for continuous variables; χ^2^ tests were used for proportions of categorical variables.

^b^Current smoker was defined as smoking at least one cigarette per day for more than half a year. Former smoker was defined as quitted smoking for more than a month.

^c^Current drinker was defined as drinking at least one time per week for more than half a year. Former drinker was defined as quitted drinking for more than a month.

^d^Physical activity was defined as exercise for at least 20 min per week for more than half a year.

^e^CVD family history was defined as CHD or stroke in a first degree relative (father, mother, siblings, or children).

^f^Hypertension was defined as self-reported physician diagnosis of hypertension, or SBP ≥140 mmHg, or DBP ≥90 mmHg, or taking antihypertensive medications.

^g^Hyperlipidemia was defined as self-reported physician diagnosis of hyperlipidemia, or TC ≥6.22 mmol/L, or TG ≥2.26 mmol/L, or LDL ≥4.14 mmol/L, or HDL < 1.04 mmol/L, or taking lipid lowering medications.

^h^Diabetes was defined as self-reported physician diagnosis of diabetes, or FG ≥7.0 mmol/L, or taking oral hypoglycemic medications or insulin.

Of 9132 participants who were included in the analyses of heart rate change, the mean (SD) age was 61 (7) years and 3838 (42.0%) were men. Most participants experienced an increase of heart rate (median 4 bpm; IQR, -3 to 11 bpm) over the mean (SD) of 4.6 (0.2) years. The distribution of change of heart rate was summarized in Suppl. Fig. 2b. Participants with increase in heart rate were more likely to be women, never smoker, never drinker, with higher prevalence of diabetes and lower baseline heart rate (Table 1).

### Association of baseline heart rate and change of heart rate with outcomes

**Figure 1:**
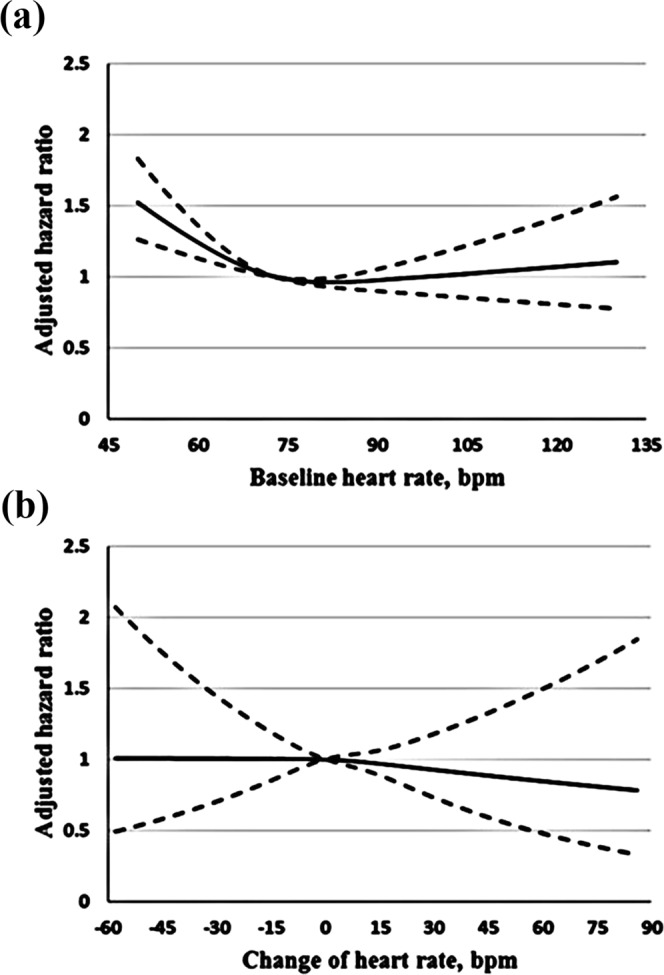
Association of baseline heart rate or change of heart rate with CVD. The adjusted cubic spline model demonstrates the flexible association of baseline heart rate with CVD (**a**), when median value of baseline heart rate is taken as the reference (73 bpm). The association of change of heart rate with CVD (**b**), when no change of heart rate is taken as the reference (0 bpm).

During a mean (SD) of 6.1 (2.4) years of follow-up, 3234 CVD events were documented among 20,828 participants. Compared with a moderate baseline heart rate, a low baseline heart rate was associated with higher risk of CVD (HR, 1.19; 95% CI, 1.07–1.32), CHD (HR, 1.22; 95% CI, 1.08–1.37) and ACS (HR, 1.34; 95% CI, 1.12–1.61), but was not associated with the risk of stroke and mortality. Low baseline heart rate was associated with higher risk of CVD (HR, 1.22; 95% CI, 1.06–1.40), CHD (HR, 1.28; 95% CI, 1.09–1.50) and ACS (HR, 1.39; 95% CI, 1.09–1.76) in men, however, the associations were not significant in women. High baseline heart rate was associated with higher risk of all-cause mortality (HR, 1.37; 95% CI, 1.17–1.61) and cardiovascular mortality (HR, 1.64; 95% CI, 1.18–2.27), but was not significantly associated with the risk of cardiovascular events (Table 2) (Suppl. Table 6). Low baseline heart rate, higher BMI, current smoking, diabetes, hypertension, hyperlipidemia was associated with higher risk of CVD. High education level was associated with lower risk of CVD (Suppl. Table 7). Restricted cubic spline plots indicated that a baseline heart rate below 65 bpm relative to a value of 73 bpm was associated with higher risk of CVD (Fig. 1a). There were no clear differences in associations of baseline heart rate with CVD according to strata of smoking status, alcohol intake status, with or without diabetes, hypertension, hyperlipidemia (Suppl. Table 8).

**Table 2:** Adjusted hazard ratios (HRs) for cardiovascular events by baseline heart rate in men and women.

Outcomes	<65 bpm	65 to 80 bpm	>80 bpm
CVD			
All			
Events/Person-years	408/13555	2262/92584	564/21109
AHR (95% CI)^a^	1.19 (1.07–1.32)	Reference	1.01 (0.92–1.11)
Men			
Events/Person-years	246/6657	1141/37078	313/9034
AHR (95% CI)^a^	1.22 (1.06–1.40)	Reference	1.06 (0.93–1.21)
Women			
Events/Person-years	162/6898	1121/55506	251/12075
AHR (95% CI)^a^	1.18 (0.99–1.39)	Reference	0.96 (0.83–1.10)
CHD			
All			
Events/Person-years	322/13786	1743/94026	439/21415
AHR (95% CI)^a^	1.22 (1.08–1.37)	Reference	1.02 (0.92–1.14)
Men			
Events/Person-years	189/6798	823/37962	234/9220
AHR (95% CI)^a^	1.28 (1.09–1.50)	Reference	1.11 (0.96–1.29)
Women			
Events/Person-years	133/6988	920/56064	205/12195
AHR (95% CI)^a^	1.16 (0.97–1.39)	Reference	0.95 (0.81–1.11)
ACS			
All			
Events/Person-years	141/13786	692/94026	183/21415
AHR (95% CI)^a^	1.34 (1.12–1.61)	Reference	1.17 (0.99–1.38)
Men			
Events/Person-years	86/6798	349/37962	105/9220
AHR (95% CI)^a^	1.39 (1.09–1.76)	Reference	1.28 (1.00–1.60)
Women			
Events/Person-years	55/6988	343/56064	78/12195
AHR (95% CI)^a^	1.29 (0.97–1.72)	Reference	1.06 (0.82–1.37)
Stroke			
All			
Events/Person-years	86/14534	519/97479	125/22269
AHR (95% CI)^a^	1.06 (0.85–1.34)	Reference	0.95 (0.78–1.17)
Men			
Events/Person-years	57/7220	318/39316	79/9646
AHR (95% CI)^a^	1.01 (0.76–1.34)	Reference	0.93 (0.72–1.19)
Women			
Events/Person-years	29/7314	201/58163	46/12623
AHR (95% CI)^a^	1.18 (0.79–1.74)	Reference	1.01 (0.73–1.41)
Ischemic stroke			
All			
Events/Person-years	71/14534	416/97479	98/22269
AHR (95% CI)^a^	1.09 (0.85–1.41)	Reference	0.95 (0.75–1.19)
Men			
Events/Person-years	49/7220	255/39316	59/9646
AHR (95% CI)^a^	1.08 (0.79–1.46)	Reference	0.87 (0.65–1.16)
Women			
Events/Person-years	22/7314	161/58163	39/12623
AHR (95% CI)^a^	1.10 (0.70–1.73)	Reference	1.10 (0.77–1.58)
Hemorrhagic stroke			
All			
Events/Person-years	15/14534	103/97479	27/22269
AHR (95% CI)^a^	0.95 (0.55–1.63)	Reference	0.98 (0.64–1.52)
Men			
Events/Person-years	8/7220	63/39316	20/9646
AHR (95% CI)^a^	0.73 (0.35–1.52)	Reference	1.15 (0.68–1.93)
Women			
Events/Person-years	7/7314	40/58163	7/12623
AHR (95% CI)^a^	1.54 (0.69–3.47)	Reference	0.70 (0.31–1.60)

Abbreviations: CVD, cardiovascular disease; CHD, coronary heart disease; ACS, acute coronary syndrome; AHR, adjusted hazard ratio.

^a^Hazard ratios were stratified for age at risk, gender, and adjusted for years of recruitment (2008–2010, 2013), BMI, smoking status, alcohol intake status, education, physical activity, hypertension, hyperlipidemia, family history of CVD, diabetes.

During a mean (SD) of 3.4 (0.7) years of follow-up, 1090 CVD events were documented among 9132 participants. Compared with a stable heart rate, an increase in heart rate was associated with higher risk of all-cause mortality (HR, 1.54; 95% CI, 1.08–2.19) and cardiovascular mortality (HR, 2.69; 95% CI, 1.43–5.05), but was not associated with the risk of cardiovascular events. Decrease in heart rate was not significantly associated with the risk of cardiovascular events and mortality. (Table 3) (Suppl. Table 9). Higher BMI, current smoking, diabetes, hypertension, hyperlipidemia was associated with higher risk of CVD (Suppl. Table 10). Restricted cubic spline plots also indicated that increase or decrease in heart rate relative to 0 bpm was not associated with risk of CVD (Fig. 1b). There were no clear differences in associations of change of heart rate with CVD according to strata of smoking status, alcohol intake status, with or without diabetes, hypertension, hyperlipidemia (Suppl. Table 11).

**Table 3:** Adjusted hazard ratios (HRs) for cardiovascular events by change of heart rate in men and women.

Outcomes	<−5 bpm	−5 to 15 bpm	>15 bpm
CVD			
All			
Events/Person-years	215/5791	712/20235	163/4814
AHR (95% CI)^a^	1.02 (0.87–1.20)	Reference	0.91 (0.77–1.09)
Men			
Events/Person-years	113/2775	339/8191	81/1870
AHR (95% CI)^a^	0.93 (0.74–1.17)	Reference	1.06 (0.83–1.36)
Women			
Events/Person-years	102/3016	373/12044	82/2944
AHR (95% CI)^a^	1.13 (0.89–1.43)	Reference	0.80 (0.63–1.02)
CHD			
All			
Events/Person-years	179/5855	584/20432	135/4847
AHR (95% CI)^a^	1.03 (0.86–1.23)	Reference	0.92 (0.76–1.12)
Men			
Events/Person-years	90/2812	265/8288	63/1897
AHR (95% CI)^a^	0.92 (0.72–1.19)	Reference	1.07 (0.81–1.41)
Women			
Events/Person-years	89/3043	319/12144	72/2950
AHR (95% CI)^a^	1.14 (0.89–1.47)	Reference	0.83 (0.64–1.07)
ACS			
All			
Events/Person-years	68/5855	181/20432	43/4847
AHR (95% CI)^a^	1.21 (0.90–1.63)	Reference	0.93 (0.66–1.30)
Men			
Events/Person-years	42/2812	86/8288	24/1897
AHR (95% CI)^a^	1.31 (0.88–1.94)	Reference	1.18 (0.75–1.88)
Women			
Events/Person-years	26/3043	95/12144	19/2950
AHR (95% CI)^a^	1.11 (0.69–1.76)	Reference	0.73 (0.44–1.21)
Stroke			
All			
Events/Person-years	36/6103	128/21308	28/5068
AHR (95% CI)^a^	0.96 (0.65–1.43)	Reference	0.89 (0.59–1.35)
Men			
Events/Person-years	23/2929	74/8636	18/1967
AHR (95% CI)^a^	0.95 (0.58–1.56)	Reference	1.05 (0.63–1.78)
Women			
Events/Person-years	13/3174	54/12672	10/3101
AHR (95% CI)^a^	0.98 (0.51–1.86)	Reference	0.72 (0.36–1.42)
Ischemic stroke			
All			
Events/Person-years	29/6103	103/21308	22/5068
AHR (95% CI)^a^	0.91 (0.59–1.42)	Reference	0.87 (0.55–1.39)
Men			
Events/Person-years	18/2929	58/8636	15/1967
AHR (95% CI)^a^	0.91 (0.52–1.60)	Reference	1.12 (0.63–1.99)
Women			
Events/Person-years	11/3174	45/12672	7/3101
AHR (95% CI)^a^	0.91 (0.45–1.86)	Reference	0.60 (0.27–1.34)
Hemorrhagic stroke			
All			
Events/Person-years	7/6103	25/21308	6/5068
AHR (95% CI)^a^	1.19 (0.49–2.91)	Reference	0.98 (0.40–2.40)
Men			
Events/Person-years	5/2929	16/8636	3/1967
AHR (95% CI)^a^	1.11 (0.38–3.24)	Reference	0.80 (0.23–2.79)
Women			
Events/Person-years	2/3174	9/12672	3/3101
AHR (95% CI)^a^	1.35 (0.27–6.75)	Reference	1.30 (0.34–4.92)

Abbreviations: CVD, cardiovascular disease; CHD, coronary heart disease; ACS, acute coronary syndrome; AHR, adjusted hazard ratio.

^a^Hazard ratios were stratified for age at risk, gender, and adjusted for baseline heart rate, BMI, smoking status, alcohol intake status, education, physical activity, hypertension, hyperlipidemia, family history of CVD, diabetes.

**Figure 2:**
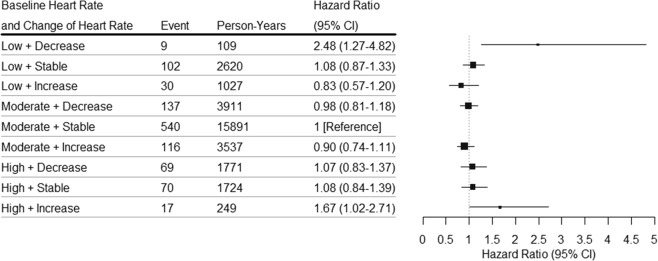
Adjusted hazard ratios (HRs) for CVD according to baseline heart rate and change of heart rate category. Baseline heart rate was categorized as low (<65 bpm), moderate (65 to 80 bpm), high (>80 bpm). Change of heart rate was categorized as decrease (more than 5 bpm), stable (−5 to 15 bpm), increase (more than 15 bpm). Hazard ratios were stratified for age at risk, gender, and adjusted for BMI, smoking status, alcohol intake status, education, physical activity, hypertension, hyperlipidemia, family history of CVD, diabetes.

Compared with stable heart rate in moderate baseline heart rate group, an increase in heart rate in high baseline heart rate group was associated with higher risk of CVD (HR, 1.67; 95% CI, 1.02–2.71), but was not associated with risk of ACS (HR, 0.78; 95% CI, 0.19–3.16). A decrease in heart rate in low baseline heart rate group was associated with higher risk of CVD (HR, 2.48; 95% CI, 1.27–4.82) and stroke (HR, 6.17; 95% CI, 2.23–17.12), but was not associated with risk of ACS (HR, 1.78; 95% CI, 0.43–7.30) (Suppl. Table 12 and Fig. 2).

## Discussion

In this large population-based cohort of the middle-aged and older Chinese, we found that low baseline heart rate was associated with higher risk of CVD, CHD and ACS, high baseline heart rate was associated with higher risk of mortality after adjustment of established confounders. Increase in heart rate was associated with higher risk of mortality, however, we did not find any significant association of change of heart rate with cardiovascular events. Both continuous increase in high baseline heart rate and decrease in low baseline heart rate were associated with higher risk of CVD.

Reduced heart rate precedes advanced conduction system, which associated with cardiovascular events^3^. The CRUSADE study, a prognostic study conducted among 139,194 patients with non-ST-segment elevation acute coronary syndromes (NSTE-ACS), showed that the lowest heart rate group (≤50 bpm) had 61% increased risk of in hospital mortality compared with the reference group (60 to 69 bpm)^6^. Consistent with the findings, we found that low baseline heart rate was associated with an elevated risk of CVD, CHD and ACS. The magnitude of risk of ACS was greater than other cardiovascular events. The CRUSADE study was focused on in hospital mortality of patients with NSTE-ACS. Our analyses confirmed and expanded these findings to a comprehensive assessment of cardiovascular events in the general population. We also found the association was stronger in men than in women, due to higher incidence of CVD and smaller variability of heart rate in men^4^. Elevated heart rate is associated with an increased risk of cardiovascular events and mortality, with increased sympathetic activity as a proposed mechanism^2^. The Framingham study indicated there was a positive association of resting heart rate with incident CVD and mortality^3^. We also found high baseline heart rate was associated with higher risk of mortality. Mao *et al*. included participants ≥40 years and free of cardiovascular disease, found that the highest heart rate group (>90 bpm) had 32% increased risk of CVD in men and 23% in women compared with the lowest heart rate group (<60 bpm)^4^. We did not find any significant association of high baseline heart rate with cardiovascular events. The average age of participants in this study was older than the Mao *et al*. studies. Given the elderly are more susceptible to chronotropic insufficiency, the associations of high baseline heart rate with cardiovascular events are likely to be weaker in the elderly^12^. The mean value of the highest heart rate group in this study (89 bpm) was lower than the value in the Mao *et al*. study (96 bpm). Another potential explanation might be there is a high threshold beyond which resting heart rate is associated with higher risk of CVD. A meta-analysis of 46 prospective cohort studies also found a significantly increased risk of cardiovascular mortality at the threshold of 90 bpm^13^.

An increase in heart rate reflects increased sympathetic activity through the development of subclinical underlying cardiovascular events, while a decrease in heart rate reflects improving cardiac function, physical fitness, and lower sympathetic tone^7^. Increase in heart rate is associated with higher risk of mortality, while the findings regarding relation of decrease in heart rate with mortality is inconsistent^7,8^. There was linear relation of change of heart rate with all-cause mortality in the ARIC study^7^. The HUNT study was conducted among 29,325 participants without known cardiovascular disease. They prospectively found that an increase in heart rate was associated with increased risk of ischemic heart disease mortality, while a decrease in heart rate showed no benefit^8^. In consistent with the HUNT study, we found increase in heart rate was associated with higher risk of cardiovascular mortality and mortality. However, we did not find any significant association of change of heart rate with cardiovascular events. As cardiovascular mortality is the worst outcome of cardiovascular events, the associations of change of heart rate with cardiovascular events may be stronger in severe cases.

Both continuous increase in high baseline heart rate (mean value from 88 bpm to 111 bpm) and decrease in low baseline heart rate (mean value from 61 bpm to 52 bpm) were associated with higher risk of CVD. There was a J-shaped relationship of heart rate and CVD. Participants of this study were general, middle-aged and older Chinese (we have excluded participants with cardiovascular disease and those taking anti-hypertensive medications), resting heart rate was relatively low. The mean value of the highest heart rate group in this study (89 bpm) was lower than the high threshold, so we did not find significant association of high baseline heart rate with CVD. The mean value of the lowest heart rate group in this study (60 bpm) was lower than the low threshold, so we found significant association of low baseline heart rate with CVD.

Current evidence is insufficient to assess whether screening resting heart rate would result in a change in risk management and ultimately reduce cardiovascular events in asymptomatic adults at intermediate or high risk of cardiovascular events^14^. Our observation adds to the evidence that monitoring and tracking resting heart rate would provide a practical approach to manage the risk of CVD in the middle-aged and older Chinese. Further studies are needed that assess the value of adding resting heart rate to CVD risk assessment tools or instruments to directly inform decision making in general population.

The mechanism of heart rate on cardiovascular events is likely to be multifactorial. On one hand, heart rate may directly contribute to cardiovascular risk. Elevated heart rate could increase hemodynamic stress and shorten the diastolic phase, which could then increase tensile stress, low and oscillatory shear stress, thereby promoting oxygen consumption; these direct detrimental effects could cause coronary atherosclerosis and myocardial ischemia^2,15^. Decreased heart rate could also cause dispersion of atrial repolarization which, in turn, initiate cardiovascular events^2^. On the other hand, heart rate may reflect other underlying processes leading to cardiovascular events. For instance, elevated heart rate reflects increased sympathetic activity^16^, which is linked to an increased risk of obesity, diabetes, hyperlipidemia and hypertension^17^.

The main strengths of this study include a novel approach is used to evaluate the joint effect of baseline heart rate and change of heart rate with CVD. This approach uses heart rate data from two surveys and relating these to cardiovascular events. To our knowledge, few studies use this approach to assess the associations of heart rate with cardiovascular events. Moreover, we split each participant’s follow up time into 5-year age-at-risk bands. The effect of age during the study (age-at-risk) is more appropriate because it determines current risk rather than age recorded at the start of the study (age-at-baseline)^18^.

There are several limitations that need to be addressed in this study. Firstly, this study is an observational study, and thus the ability to make causal inferences is limited. Secondly, it is lack of information on medications or other medical treatment which would influence heart rate. Given that we have excluded participants with cardiovascular disease and those taking anti-hypertensive medications, these confounding factors may show minor influence on the results. Thirdly, some studies use the time updated approach to calculate actual change in heart rate by repeated measurement^7^, while, we only assess heart rate twice. Finally, ultrasound echocardiography was not performed at two surveys. Given that we have excluded most heart diseases that can affect heart rate. Furthermore, participants with suspected CHD undertook ultrasound echocardiography at hospital to help diagnosis. So, it would not dramatically affect the results of this study.

In conclusion, low resting heart rate is associated with higher risk of CVD. Both continuous increase in high baseline heart rate and decrease in low baseline heart rate are associated with higher risk of CVD. The availability of easily monitoring and tracking resting heart rate would provide a practical approach to manage the risk of CVD in the middle-aged and older Chinese.

## Methods

### Study population

The design of Dongfeng-Tongji (DFTJ) cohort has been described previously^19^. In brief, a total of 27,009 retired employees were enrolled in the baseline survey, completed questionnaire interview and clinical measurements between September 2008 and June 2010. The resurvey in 2013 enrolled 38,295 participants, while 24,175 of them participated in both surveys (overall response rate was 90%) and 14,120 of them were newly enrolled in the resurvey. They completed questionnaire interview and clinical measurements as those in the baseline survey. The mean (SD) of time interval between two surveys was 4.6 (0.2) years.

Among the 27,009 participants who were enrolled in the baseline survey, a total of 12,904 participants were excluded due to the following reasons: loss to follow-up (n = 709), self-reported of physician diagnosed CHD (n = 4739), cancer (n = 1631), stroke (n = 1472), with atrial fibrillation (n = 242), atrial flutter (n = 5), pre-excitation syndrome (n = 14), pacemaker rhythm (n = 32), frequent premature ventricular contractions (n = 22), missing information on heart rate (n = 868), with anti-hypertensive medications (n = 4773), and heart rate >130 bpm (n = 8). Among the 14,120 participants who were newly enrolled in the resurvey, a total of 7397 participants were excluded due to the following reasons: self-reported of physician diagnosed CHD (n = 991), cancer (n = 551), stroke (n = 500), with atrial fibrillation (n = 76), atrial flutter (n = 2), pre-excitation syndrome (n = 6), pacemaker rhythm (n = 7), frequent premature ventricular contractions (n = 6), missing information on heart rate (n = 3255), with anti-hypertensive medications (n = 3099), and heart rate >130 bpm (n = 29). Therefore, 20,828 participants were included in the analyses of baseline heart rate (Suppl. Fig. 1a). The excluded participants had higher prevalence of hypertension, diabetes and hyperlipidemia than those who were included (P < 0.05) (Suppl. Table 1). Participants with anti-hypertensive medications had higher baseline heart rate than those without anti-hypertensive medications (P < 0.05) (Suppl. Table 2).

Among the 24,175 participants who participated in both the baseline survey and the resurvey, a total of 15,043 participants were excluded due to the following reasons: diagnosed CHD (n = 5773), cancer (n = 2047), stroke (n = 1906), with atrial fibrillation (n = 310), atrial flutter (n = 7), pre-excitation syndrome (n = 16), pacemaker rhythm (n = 57), frequent premature ventricular contractions (n = 44), missing information on heart rate (n = 4248), with anti-hypertensive medications (n = 3572), and baseline heart rate >130 bpm (n = 3). Therefore, 9132 participants were included in the analyses of heart rate change (Suppl. Fig. 1b). The excluded participants had higher prevalence of hypertension, diabetes and hyperlipidemia and higher baseline heart rate than those who were included (P < 0.05) (Suppl. Table 3).

The study was approved by the Ethics and Human Subject Committees of the Tongji Medical College. All the methods in the present study were carried out in accordance with the approved guidelines. All participants provided written informed consents.

### Baseline heart rate and calculation of change of heart rate

Resting heart rate was measured using standard supine 12-lead electrocardiography (Cardiofax ECG-9020P), which was performed after approximately 5 minutes of rest. Baseline heart rate was the first available heart rate value for each participant over the course of study. Change of heart rate was calculated by subtracting the heart rate value in the resurvey from the value in the baseline survey. Baseline heart rate was categorized as low (<65 bpm), moderate (65 to 80 bpm), high (>80 bpm), according to integral values near 20th and 80th percentiles. Change of heart rate was categorized as decrease (more than 5 bpm), stable (-5 to 15 bpm), increase (more than 15 bpm), according to integral values near 20th and 80th percentiles.

### Ascertainment of outcomes

We tracked participants’ morbidity and mortality records through health-care service system. An expert panel of physicians reviewed medical insurance documents, hospital records, and death certificates from the baseline survey to December 31, 2016 and confirmed cardiovascular events and mortality based on diagnosis criteria and International Classification of Diseases (ICD) codes of the WHO^20^. The number of CVD were calculated from the number of CHD or stroke, whichever came first. CHD was defined as stenosis ≥50% in at least one major coronary artery or death with CHD as the underlying cause (ICD-10 codes I20-I25)^21,22^. ACS was severe type of CHD, including acute myocardial infarction (ICD-10 codes I21.0-I21.4) and unstable angina (ICD-10 codes I20.0, I20.1, I20.9)^23^. Stroke was defined as sudden or rapid onset of a typical neurological deficit of vascular origin that persisted more than 24 hours or death with stroke as the underlying cause (ICD-10 codes I60, I61, I63, I64, I69.0, I69.1, I69.3, I69.4), including ischemic stroke and hemorrhagic stroke^24^. Cardiovascular events included CVD, CHD, ACS, ischemic stroke, and hemorrhagic stroke. Cardiovascular mortality was defined as death with CVD (ICD-10: I00–I99).

### Statistical analysis

Basic characteristics of the study population were described as frequencies with percentages for categorical variables and means with SDs or medians with interquartile ranges (IQRs) for continuous variables. Statistical differences between groups were compared using linear regression analyses for continuous variables and χ^2^ tests for categorical variables. The associations of baseline heart rate and change of heart rate with outcomes were assessed with multivariable Cox proportional hazards models. The multivariable models were stratified for age at risk (in 5-year intervals)^18^, gender, and adjusted for established risk factors of CVD, including education, smoking status, alcohol intake status, physical activity, family history of CVD, BMI, hypertension, hyperlipidemia, diabetes. In addition, we adjusted for baseline heart rate when modeling for change of heart rate. Missing data of covariates were handled by following imputation approaches. For continuous covariates, an extra binary variable identifying which observation on that data were missing was additionally adjusted and the missing values were replaced with the median of the observed values for that variable. For categorical covariates, an extra category was added indicating missingness^25^. Proportional hazards assumptions were checked by Schoenfeld residuals. Results were presented as hazard ratios (HRs) with 95% confidence intervals (CIs). We used the restricted cubic spline with 4 knots (20th, 40th, 60th, 80th) to flexibly display the association between baseline heart rate and the hazards of developing cardiovascular event, with a reference value set at 73 bpm. For the change of heart rate, 0 bpm was used as the reference. We further conducted subgroup analyses to determine whether the associations of baseline heart rate or change of heart rate with cardiovascular events varied in different subgroups (smoking status, alcohol intake status, with or without diabetes, hypertension, hyperlipidemia). Finally, we categorized change of heart rate by baseline heart rate to assess the joint effect of baseline heart rate and change of heart rate with cardiovascular events. Two-sided *P* values were used and *P* < 0.05 denoted statistical significance. All analyses were performed using SAS version 9.3 (SAS Institute Inc), and R version 3.4.2 (R Foundation).

## Supplementary information

SUPPLEMENTARY INFO

## Data Availability

The datasets generated during and/or analysed during the current study are available from the corresponding author on reasonable request.
