# Supplementary material for: Association of resting heart rate and its change with incident cardiovascular events in the middle-aged and older Chinese
Source: Sci Rep. 2019 Apr 25;9:6556. doi: 10.1038/s41598-019-43045-5 (PMC6484081; doi:10.1038/s41598-019-43045-5)

**Supplementary Information**

**Association of resting heart rate and its change with incident cardiovascular events in the middle-aged and older Chinese**

Jing Tian, Yu Yuan, Miaoyan Shen, Xiaomin Zhang, Meian He, Huan Guo, Handong Yang, Tangchun Wu

| **Supplementary Table 1.** Comparison of participants included in the analyses of baseline heart rate with those who were excluded | | | |
| --- | --- | --- | --- |
| **Variables** | **Included** | **Excluded** | ***P* Value ^a^** |
| No. of participants | 20828 | 20301 |  |
| Age, mean (SD), years | 60.8 (7.9) | 64.4 (8.1) | <0.01 |
| Men, No. (%) | 8805 (42.3) | 9730 (47.9) | <0.01 |
| BMI, mean (SD), kg/m^2^ | 23.8 (3.1) | 24.8 (3.1) | <0.01 |
| Smoking status, No. (%) ^b^ |  |  |  |
| Current smoker | 3970 (19.1) | 3545 (17.5) | <0.01 |
| Former smoker | 1831 (8.8) | 2886 (14.2) |  |
| Never smoker | 14910 (71.6) | 13763 (67.8) |  |
| Alcohol intake status, No. (%) ^c^ |  |  |  |
| Current drinker | 5134 (24.6) | 4225 (20.8) | <0.01 |
| Former drinker | 812 (3.9) | 1538 (7.6) |  |
| Never drinker | 14849 (71.3) | 14499 (71.4) |  |
| Education level, No. (%) |  |  |  |
| Primary school or below | 4646 (22.3) | 5010 (24.7) | <0.01 |
| Middle school | 7635 (36.7) | 6955 (34.3) |  |
| High school or beyond | 8392 (40.3) | 8191 (40.3) |  |
| Physical activity (yes), No. (%) ^d^ | 14584 (70.0) | 14223 (70.1) | 0.70 |
| CVD family history, No. (%) ^e^ | 2282 (11.0) | 2748 (13.5) | 0.33 |
| Hypertension, No. (%) ^f^ | 7355 (35.3) | 14997 (73.9) | <0.01 |
| Hyperlipidemia, No. (%) ^g^ | 7246 (34.8) | 10558 (52.0) | <0.01 |
| Diabetes, No. (%) ^h^ | 2591 (12.4) | 4859 (23.9) | <0.01 |
| Baseline heart rate, mean (SD), bpm | 74.7 (9.7) | 74.8 (10.8) | 0.10 |

Abbreviations: BMI, body mass index; CVD, cardiovascular disease.

^a^ Mann-Whitney U tests for continuous variables, and Chi-square tests for categorical variables.

^b^ Current smoker was defined as smoking at least one cigarette per day for more than half a year. Former smoker was defined as quitted smoking for more than a month.

^c^ Current drinker was defined as drinking at least one time per week for more than half a year. Former drinker was defined as quitted drinking for more than a month.

^d^ Physical activity was defined as exercise for at least 20 min per week for more than half a year.

^e^ CVD family history was defined as CHD or stroke in a first degree relative (father, mother, siblings, or children).

^f^ Hypertension was defined as self-reported physician diagnosis of hypertension, or SBP≥140 mmHg, or DBP≥90 mmHg, or taking antihypertensive medications.

^g^ Hyperlipidemia was defined as self-reported physician diagnosis of hyperlipidemia, or TC ≥ 6.22 mmol/L, or TG ≥ 2.26 mmol/L, or LDL ≥ 4.14 mmol/L, or HDL < 1.04 mmol/L, or taking lipid lowering medications.

^h^ Diabetes was defined as self-reported physician diagnosis of diabetes, or FG ≥7.0 mmol/L, or taking oral hypoglycemic medications or insulin.

| **Supplementary Table 2**. Comparison of participants with or without anti-hypertensive medications | | | |
| --- | --- | --- | --- |
| **Variables** | **Participants with anti-hypertensive medications** | **Participants without anti-hypertensive medications** | ***P* Value ^a^** |
| No. of participants | 6800 | 20828 |  |
| Age, mean (SD), years | 64.3 (7.5) | 60.8 (7.9) | <0.01 |
| Men, No. (%) | 3217 (47.3) | 8805 (42.3) | <0.01 |
| BMI, mean (SD), kg/m^2^ | 25.4 (3.3) | 23.8 (3.1) | <0.01 |
| Smoking status, No. (%) ^b^ |  |  |  |
| Current smoker | 1181 (17.4) | 3970 (19.1) | <0.01 |
| Former smoker | 863 (12.7) | 1831 (8.8) |  |
| Never smoker | 4726 (69.5) | 14910 (71.6) |  |
| Alcohol intake status, No. (%) ^c^ |  |  |  |
| Current drinker | 1539 (22.6) | 5134 (24.6) | <0.01 |
| Former drinker | 409 (6.0) | 812 (3.9) |  |
| Never drinker | 4845 (71.3) | 14849 (71.3) |  |
| Education level, No. (%) |  |  |  |
| Primary school or below | 1745 (25.7) | 4646 (22.3) | <0.01 |
| Middle school | 2437 (35.8) | 7635 (36.7) |  |
| High school or beyond | 2584 (38.0) | 8392 (40.3) |  |
| Physical activity (yes), No. (%) ^d^ | 5007 (73.6) | 14584 (70.0) | <0.01 |
| CVD family history, No. (%) ^e^ | 713 (10.5) | 2282 (11.0) | 0.28 |
| Hyperlipidemia, No. (%) ^f^ | 3912 (57.5) | 7246 (34.8) | <0.01 |
| Diabetes, No. (%) ^g^ | 1844 (27.1) | 2591 (12.4) | <0.01 |
| Baseline heart rate, mean (SD), bpm | 75.4 (10.9) | 74.7 (9.7) | <0.01 |

Abbreviations: BMI, body mass index; CVD, cardiovascular disease.

^a^ Mann-Whitney U tests for continuous variables, and Chi-square tests for categorical variables.

^b^ Current smoker was defined as smoking at least one cigarette per day for more than half a year. Former smoker was defined as quitted smoking for more than a month.

^c^ Current drinker was defined as drinking at least one time per week for more than half a year. Former drinker was defined as quitted drinking for more than a month.

^d^ Physical activity was defined as exercise for at least 20 min per week for more than half a year.

^e^ CVD family history was defined as CHD or stroke in a first degree relative (father, mother, siblings, or children).

^f^ Hyperlipidemia was defined as self-reported physician diagnosis of hyperlipidemia, or TC ≥ 6.22 mmol/L, or TG ≥ 2.26 mmol/L, or LDL ≥ 4.14 mmol/L, or HDL < 1.04 mmol/L, or taking lipid lowering medications.

^g^ Diabetes was defined as self-reported physician diagnosis of diabetes, or FG ≥7.0 mmol/L, or taking oral hypoglycemic medications or insulin.

| **Supplementary Table 3.** Comparison of participants included in the analyses of change of heart rate with those who were excluded | | | |
| --- | --- | --- | --- |
| **Variables** | **Included** | **Excluded** | ***P* Value ^a^** |
| No. of participants | 9132 | 15043 |  |
| Age, mean (SD), years | 61.2 (7.3) | 64.4 (7.5) | <0.01 |
| Men, No. (%) | 3838 (42.0） | 6778 (45.1) | 0.15 |
| BMI, mean (SD), kg/m^2^ | 23.9 (3.1) | 24.9 (3.3) | <0.01 |
| Smoking status, No. (%) ^b^ |  |  |  |
| Current smoker | 1705 (18.7) | 2522 (16.8) | <0.01 |
| Former smoker | 786 (8.6) | 1962 (13.0) |  |
| Never smoker | 6599 (72.3) | 10424 (69.3) |  |
| Alcohol intake status, No. (%) ^c^ |  |  |  |
| Current drinker | 2121 (23.2) | 2950 (19.6) | <0.01 |
| Former drinker | 338 (3.7) | 985 (6.5) |  |
| Never drinker | 6668 (73.0) | 11087 (73.7) |  |
| Education level, No. (%) |  |  |  |
| Primary school or below | 2589 (28.4) | 4419 (29.4) | <0.01 |
| Middle school | 3386 (37.1) | 5370 (35.7) |  |
| High school or beyond | 3083 (33.8) | 5139 (34.2) |  |
| Physical activity (yes), No. (%) ^d^ | 6470 (70.8) | 10752 (71.5) | 0.92 |
| CVD family history, No. (%) ^e^ | 790 (8.7) | 1737 (11.5) | 0.83 |
| Hypertension, No. (%) ^f^ | 2907 (31.8) | 10275 (68.3) | <0.01 |
| Hyperlipidemia, No. (%) ^g^ | 3301 (36.1) | 7509 (49.9) | <0.01 |
| Diabetes, No. (%) ^h^ | 1149 (12.6) | 3195 (21.2) | <0.01 |
| Baseline heart rate, mean (SD), bpm | 72.8 (8.9) | 73.9 (9.5) | <0.01 |

Abbreviations: BMI, body mass index; CVD, cardiovascular disease.

^a^ Mann-Whitney U tests for continuous variables, and Chi-square tests for categorical variables.

^b^ Current smoker was defined as smoking at least one cigarette per day for more than half a year. Former smoker was defined as quitted smoking for more than a month.

^c^ Current drinker was defined as drinking at least one time per week for more than half a year. Former drinker was defined as quitted drinking for more than a month.

^d^ Physical activity was defined as exercise for at least 20 min per week for more than half a year.

^e^ CVD family history was defined as CHD or stroke in a first degree relative (father, mother, siblings, or children).

^f^ Hypertension was defined as self-reported physician diagnosis of hypertension, or SBP≥140 mmHg, or DBP≥90 mmHg, or taking antihypertensive medications.

^g^ Hyperlipidemia was defined as self-reported physician diagnosis of hyperlipidemia, or TC ≥ 6.22 mmol/L, or TG ≥ 2.26 mmol/L, or LDL ≥ 4.14 mmol/L, or HDL < 1.04 mmol/L, or taking lipid lowering medications.

^h^ Diabetes was defined as self-reported physician diagnosis of diabetes, or FG ≥7.0 mmol/L, or taking oral hypoglycemic medications or insulin.

| **Supplementary Table 4.** Correlations between resting heart rate and influencing factors | | |
| --- | --- | --- |
| **Variables** | **r ^a^** | ***P* Value** |
| Age (years) | -0.05 | <0.01 |
| Body mass index (kg/m^2^) | -0.04 | <0.01 |
| Systolic blood pressure (mm Hg) | 0.14 | <0.01 |
| Diastolic blood pressure (mm Hg) | 0.17 | <0.01 |
| Fasting blood glucose (mmol/L) | 0.08 | <0.01 |
| Total cholesterol (mmol/L) | 0.02 | 0.01 |
| Triglyceride (mmol/L) | 0.08 | <0.01 |
| High-density lipoprotein cholesterol (mmol/L) | -0.01 | 0.69 |
| Low-density lipoprotein cholesterol (mmol/L) | -0.01 | 0.08 |

^a^ Spearman’s rank correlation coefficients were shown in the table.

| **Supplementary Table 5.** Linear regression analysis of influencing factors of resting heart rate | | | |
| --- | --- | --- | --- |
| **Variables** | **β ^a^** | **SE** | ***P* Value** |
| Age (years) | -0.07 | 0.01 | <0.01 |
| Men | -0.33 | 0.16 | 0.04 |
| Body mass index (kg/m^2^) | -0.30 | 0.02 | <0.01 |
| Current smoker (Yes) | -0.11 | 0.09 | 0.23 |
| Current drinker (Yes) | -0.10 | 0.13 | 0.43 |
| Physical activity (Yes) | -0.49 | 0.14 | <0.01 |
| Systolic blood pressure (mm Hg) | 0.05 | 0.01 | <0.01 |
| Diastolic blood pressure (mm Hg) | 0.11 | 0.01 | <0.01 |
| Fasting blood glucose (mmol/L) | 0.54 | 0.04 | <0.01 |
| Total cholesterol (mmol/L) | -0.22 | 0.13 | 0.11 |
| Triglyceride (mmol/L) | 0.35 | 0.07 | <0.01 |
| High-density lipoprotein cholesterol (mmol/L) | 0.07 | 0.20 | 0.73 |
| Low-density lipoprotein cholesterol (mmol/L) | 0.18 | 0.14 | 0.19 |

^a^ Multivariate linear regression model included age, gender, BMI, smoking status, alcohol intake status, physical activity, systolic blood pressure, diastolic blood pressure, fasting blood glucose, total cholesterol, triglyceride, high-density lipoprotein cholesterol, low-density lipoprotein cholesterol.

| **Supplementary Table 6.** Adjusted hazard ratios (HRs) for mortality by baseline heart rate | | | |
| --- | --- | --- | --- |
| **Outcomes** | **<65 bpm** | **65 to 80 bpm** | **>80 bpm** |
| All-cause mortality |  |  |  |
| Events | 106 | 669 | 217 |
| AHR (95% CI) ^a^ | 0.95 (0.78-1.17) | Reference | 1.37 (1.17-1.61) |
| Cardiovascular mortality |  |  |  |
| Events | 24 | 127 | 56 |
| AHR (95% CI) ^a^ | 1.13 (0.73-1.76) | Reference | 1.64 (1.18-2.27) |

Abbreviations: AHR, adjusted hazard ratio.

^a^ Hazard ratios were stratified for age at risk, gender, and adjusted for years of recruitment (2008-2010, 2013), BMI, smoking status, alcohol intake status, education, physical activity, hypertension, hyperlipidemia, family history of CVD, diabetes.

| **Supplementary Table 7.** Cox proportional hazard model for cardiovascular events by baseline heart rate | | | | | | | | |  |
| --- | --- | --- | --- | --- | --- | --- | --- | --- | --- |
| **Variables** | **CVD** | |  | **CHD** | |  | **Stroke** | | |
|  | **AHR (95% CI) ^a^** | ***P* Value** |  | **AHR (95% CI) ^a^** | ***P* Value** |  | **AHR (95% CI) ^a^** | ***P* Value** | |
| Baseline heart rate, bpm |  |  |  |  |  |  |  |  | |
| <65 bpm | 1.19 (1.07-1.32) | <0.01 |  | 1.22 (1.08-1.37) | <0.01 |  | 1.06 (0.85-1.34) | 0.59 | |
| 65-80 bpm | Reference | - |  | Reference | - |  | Reference | - | |
| >80 bpm | 1.01 (0.92-1.11) | 0.90 |  | 1.02 (0.92-1.14) | 0.68 |  | 0.95 (0.78-1.17) | 0.64 | |
| BMI, kg/m^2^ | 1.01 (1.00-1.02) | 0.02 |  | 1.01 (1.00-1.03) | 0.03 |  | 1.01 (0.99-1.03) | 0.45 | |
| Smoking status |  |  |  |  |  |  |  |  | |
| Current smoker | 1.30 (1.16-1.44) | <0.01 |  | 1.23 (1.08-1.39) | <0.01 |  | 1.43 (1.16-1.76) | <0.01 | |
| Former smoker | 1.20 (1.05-1.36) | 0.01 |  | 1.23 (1.06-1.43) | 0.01 |  | 1.05 (0.80-1.36) | 0.74 | |
| Never smoker | Reference | - |  | Reference | - |  | Reference | - | |
| Alcohol intake status |  |  |  |  |  |  |  |  | |
| Current drinker | 0.95 (0.87-1.04) | 0.28 |  | 0.94 (0.85-1.05) | 0.28 |  | 0.99 (0.82-1.19) | 0.90 | |
| Former drinker | 0.75 (0.62-0.91) | <0.01 |  | 0.72 (0.58-0.90) | <0.01 |  | 0.90 (0.63-1.30) | 0.57 | |
| Never drinker | Reference | - |  | Reference | - |  | Reference | - | |
| Education level |  |  |  |  |  |  |  |  | |
| Primary school or below | Reference | - |  | Reference | - |  | Reference | - | |
| Middle school | 1.02 (0.93-1.11) | 0.72 |  | 1.04 (0.94-1.15) | 0.41 |  | 0.93 (0.78-1.10) | 0.39 | |
| High school or beyond | 0.82 (0.75-0.90) | <0.01 |  | 0.89 (0.80-0.99) | 0.04 |  | 0.62 (0.51-0.76) | <0.01 | |
| Physical activity |  |  |  |  |  |  |  |  | |
| Yes | 1.01 (0.93-1.09) | 0.90 |  | 1.01 (0.92-1.10) | 0.84 |  | 0.99 (0.85-1.17) | 0.94 | |
| No | Reference | - |  | Reference | - |  | Reference | - | |

| **Supplementary Table 7.** Cox proportional hazard model for cardiovascular events by baseline heart rate (continued) | | | | | | | | |
| --- | --- | --- | --- | --- | --- | --- | --- | --- |
| **Variables** | **CVD** | |  | **CHD** | |  | **Stroke** | |
|  | **AHR (95% CI) ^a^** | ***P* Value** |  | **AHR (95% CI) ^a^** | ***P* Value** |  | **AHR (95% CI) ^a^** | ***P* Value** |
| Family history of CVD |  |  |  |  |  |  |  |  |
| Yes | 0.97 (0.85-1.11) | 0.70 |  | 1.05 (0.91-1.22) | 0.47 |  | 0.65 (0.45-0.93) | 0.02 |
| No | Reference | - |  | Reference | - |  | Reference | - |
| Hypertension |  |  |  |  |  |  |  |  |
| Yes | 1.38 (1.28-1.48) | <0.01 |  | 1.24 (1.14-1.35) | <0.01 |  | 1.86 (1.60-2.16) | <0.01 |
| No | Reference | - |  | Reference | - |  | Reference | - |
| Hyperlipidemia |  |  |  |  |  |  |  |  |
| Yes | 1.31 (1.22-1.41) | <0.01 |  | 1.35 (1.24-1.46) | <0.01 |  | 1.15 (0.99-1.34) | 0.06 |
| No | Reference | - |  | Reference | - |  | Reference | - |
| Diabetes |  |  |  |  |  |  |  |  |
| Yes | 1.37 (1.25-1.50) | <0.01 |  | 1.33 (1.20-1.47) | <0.01 |  | 1.47 (1.22-1.77) | <0.01 |
| No | Reference | - |  | Reference | - |  | Reference | - |

Abbreviations: CVD, cardiovascular disease; CHD, coronary heart disease; AHR, adjusted hazard ratio.

^a^ Cox proportional hazard model were stratified for age at risk, gender, and included baseline heart rate, years of recruitment (2008-2010, 2013), BMI, smoking status, alcohol intake status, education, physical activity, hypertension, hyperlipidemia, family history of CVD, diabetes.

| **Supplementary Table 8.** Adjusted hazard ratios (HRs) for CVD by baseline heart rate stratified by risk factors | | | |
| --- | --- | --- | --- |
| **Subgroup** | **<65 bpm** | **65-80 bpm** | **>80 bpm** |
| Smoking status |  |  |  |
| Current smoker |  |  |  |
| Events/Person-years | 104/3091 | 535/16450 | 128/3988 |
| AHR (95% CI) ^a^ | 1.05 (0.85 to 1.30) | Reference | 0.97 (0.80 to 1.19) |
| Former smoker |  |  |  |
| Events/Person-years | 51/1297 | 259/7659 | 75/1935 |
| AHR (95% CI) ^a^ | 1.20 (0.88 to 1.63) | Reference | 1.02 (0.78 to 1.34) |
| Never smoker |  |  |  |
| Events/Person-years | 253/9146 | 1460/67835 | 361/15121 |
| AHR (95% CI) ^a^ | 1.26 (1.10 to 1.44) | Reference | 1.02 (0.90 to 1.15) |
| Alcohol intake status |  |  |  |
| Current drinker |  |  |  |
| Events/Person-years | 115/3512 | 578/21283 | 161/5354 |
| AHR (95% CI) ^a^ | 1.19 (0.98 to 1.46) | Reference | 1.06 (0.88 to 1.27) |
| Former drinker |  |  |  |
| Events/Person-years | 20/447 | 78/3681 | 26/765 |
| AHR (95% CI) ^a^ | 2.00 (1.20 to 3.34) | Reference | 1.71 (1.06 to 2.75) |
| Never drinker |  |  |  |
| Events/Person-years | 272/9591 | 1600/67497 | 377/14960 |
| AHR (95% CI) ^a^ | 1.16 (1.02 to 1.32) | Reference | 0.97 (0.86 to 1.09) |
| Diabetes |  |  |  |
| YES |  |  |  |
| Events/Person-years | 69/1380 | 384/10376 | 146/3354 |
| AHR (95% CI) ^a^ | 1.19 (0.91 to 1.54) | Reference | 1.12 (0.91 to1.37) |
| NO |  |  |  |
| Events/Person-years | 339/12175 | 1878/82208 | 418/17754 |
| AHR (95% CI) ^a^ | 1.19 (1.06 to 1.34) | Reference | 0.97 (0.87 to 1.08) |
| Hypertension |  |  |  |
| YES |  |  |  |
| Events/Person-years | 153/3358 | 1007/30036 | 317/9488 |
| AHR (95% CI) ^a^ | 1.28 (1.08 to 1.52) | Reference | 0.99 (0.87 to 1.12) |
| NO |  |  |  |
| Events/Person-years | 255/10196 | 1255/62547 | 247/11620 |
| AHR (95% CI) ^a^ | 1.16 (1.01 to 1.33) | Reference | 1.03 (0.90 to 1.19) |
| Hyperlipidemia |  |  |  |
| YES |  |  |  |
| Events/Person-years | 175/4312 | 956/31501 | 272/7785 |
| AHR (95% CI) ^a^ | 1.28 (1.09 to 1.50) | Reference | 1.07 (0.93 to 1.23) |
| NO |  |  |  |
| Events/Person-years | 233/9242 | 1306/61082 | 292/13324 |
| AHR (95% CI) ^a^ | 1.14 (0.99 to 1.31) | Reference | 0.95 (0.84 to 1.08) |

Abbreviations: CVD, cardiovascular disease; AHR, adjusted hazard ratio.

^a^ Hazard ratios were stratified for age at risk, gender, and adjusted for years of recruitment (2008-2010, 2013), BMI, smoking status, alcohol intake status, education, physical activity, hypertension, hyperlipidemia, family history of CVD, diabetes, except for the stratified variable itself.

| **Supplementary Table 9.** Adjusted hazard ratios (HRs) for mortality by change of heart rate | | | |
| --- | --- | --- | --- |
| **Outcomes** | **< -5 bpm** | **-5 to 15 bpm** | **>15 bpm** |
| All-cause mortality |  |  |  |
| Events | 48 | 124 | 44 |
| AHR (95% CI) ^a^ | 0.96 (0.67-1.38) | Reference | 1.54 (1.08-2.19) |
| Cardiovascular mortality |  |  |  |
| Events | 10 | 29 | 16 |
| AHR (95% CI) ^a^ | 0.73 (0.34-1.57) | Reference | 2.69 (1.43-5.05) |

Abbreviations: AHR, adjusted hazard ratio.

^a^ Hazard ratios were stratified for age at risk, gender, and adjusted for baseline heart rate, BMI, smoking status, alcohol intake status, education, physical activity, hypertension, hyperlipidemia, family history of CVD, diabetes.

| **Supplementary Table 10.** Cox proportional hazard model for cardiovascular events by change of heart rate | | | | | | | | |
| --- | --- | --- | --- | --- | --- | --- | --- | --- |
| **Variables** | **CVD** | |  | **CHD** | |  | **Stroke** | |
|  | **AHR (95% CI) ^a^** | ***P* Value** |  | **AHR (95% CI) ^a^** | ***P* Value** |  | **AHR (95% CI) ^a^** | ***P* Value** |
| Change of heart rate, bpm |  |  |  |  |  |  |  |  |
| <-5 bpm | 1.02 (0.87-1.20) | 0.81 |  | 1.03 (0.86-1.23) | 0.77 |  | 0.96 (0.65-1.43) | 0.85 |
| -5 to 15 bpm | Reference | - |  | Reference | - |  | Reference | - |
| >15 bpm | 0.91 (0.77-1.09) | 0.30 |  | 0.92 (0.76-1.12) | 0.42 |  | 0.89 (0.59-1.35) | 0.59 |
| Baseline heart rate, bpm | 1.00 (0.99-1.01) | 0.83 |  | 1.00 (0.99-1.01) | 0.89 |  | 0.99 (0.98-1.01) | 0.48 |
| BMI, kg/m^2^ | 1.03 (1.01-1.05) | <0.01 |  | 1.03 (1.01-1.05) | <0.01 |  | 1.02 (0.98-1.07) | 0.33 |
| Smoking status |  |  |  |  |  |  |  |  |
| Current smoker | 1.24 (1.03-1.50) | 0.02 |  | 1.21 (0.97-1.49) | 0.09 |  | 1.33 (0.88-2.00) | 0.18 |
| Former smoker | 1.14 (0.90-1.44) | 0.28 |  | 1.15 (0.89-1.49) | 0.28 |  | 1.05 (0.62-1.79) | 0.85 |
| Never smoker | Reference | - |  | Reference | - |  | Reference | - |
| Alcohol intake status |  |  |  |  |  |  |  |  |
| Current drinker | 0.98 (0.83-1.15) | 0.79 |  | 1.00 (0.83-1.20) | 0.98 |  | 0.92 (0.64-1.33) | 0.65 |
| Former drinker | 0.62 (0.43-0.90) | 0.01 |  | 0.59 (0.39-0.90) | 0.01 |  | 0.79 (0.37-1.67) | 0.54 |
| Never drinker | Reference | - |  | Reference | - |  | Reference | - |
| Education level |  |  |  |  |  |  |  |  |
| Primary school or below | Reference | - |  | Reference | - |  | Reference | - |
| Middle school | 1.01 (0.87-1.17) | 0.88 |  | 1.07 (0.91-1.26) | 0.40 |  | 0.77 (0.55-1.08) | 0.13 |
| High school or beyond | 0.88 (0.75-1.03) | 0.11 |  | 0.95 (0.79-1.13) | 0.54 |  | 0.63 (0.43-0.93) | 0.02 |
| Physical activity |  |  |  |  |  |  |  |  |
| Yes | 1.10 (0.96-1.26) | 0.16 |  | 1.14 (0.98-1.33) | 0.09 |  | 0.94 (0.68-1.28) | 0.69 |
| No | Reference | - |  | Reference | - |  | Reference | - |

| **Supplementary Table 10.** Cox proportional hazard model for cardiovascular events by change of heart rate (continued) | | | | | | | | |
| --- | --- | --- | --- | --- | --- | --- | --- | --- |
| **Variables** | **CVD** | |  | **CHD** | |  | **Stroke** | |
|  | **AHR (95% CI) ^a^** | ***P* Value** |  | **AHR (95% CI) ^a^** | ***P* Value** |  | **AHR (95% CI) ^a^** | ***P* Value** |
| Family history of CVD |  |  |  |  |  |  |  |  |
| Yes | 0.77 (0.59-1.01) | 0.05 |  | 0.81 (0.61-1.08) | 0.15 |  | 0.59 (0.27-1.26) | 0.17 |
| No | Reference | - |  | Reference | - |  | Reference | - |
| Hypertension |  |  |  |  |  |  |  |  |
| Yes | 1.25 (1.10-1.42) | <0.01 |  | 1.15 (1.00-1.32) | 0.05 |  | 1.79 (1.34-2.41) | <0.01 |
| No | Reference | - |  | Reference | - |  | Reference | - |
| Hyperlipidemia |  |  |  |  |  |  |  |  |
| Yes | 1.26 (1.11-1.42) | <0.01 |  | 1.29 (1.13-1.48) | <0.01 |  | 1.07 (0.79-1.44) | 0.67 |
| No | Reference | - |  | Reference | - |  | Reference | - |
| Diabetes |  |  |  |  |  |  |  |  |
| Yes | 1.36 (1.16-1.59) | <0.01 |  | 1.35 (1.13-1.60) | <0.01 |  | 1.37 (0.94-1.99) | 0.10 |
| No | Reference | - |  | Reference | - |  | Reference | - |

Abbreviations: CVD, cardiovascular disease; CHD, coronary heart disease; AHR, adjusted hazard ratio.

^a^ Cox proportional hazard model were stratified for age at risk, gender, and included change of heart rate, baseline heart rate, BMI, smoking status, alcohol intake status, education, physical activity, hypertension, hyperlipidemia, family history of CVD, diabetes.

| **Supplementary Table 11.** Adjusted hazard ratios (HRs) for CVD by change of heart rate stratified by risk factors | | | |
| --- | --- | --- | --- |
| **Subgroup** | **<-5 bpm** | **-5 to 15 bpm** | **>15 bpm** |
| Smoking status |  |  |  |
| Current smoker |  |  |  |
| Events/Person-years | 53/1277 | 150/3622 | 38/763 |
| AHR (95% CI) ^a^ | 0.97 (0.69 to 1.35) | Reference | 1.22 (0.84 to 1.75) |
| Former smoker |  |  |  |
| Events/Person-years | 23/552 | 74/1691 | 18/381 |
| AHR (95% CI) ^a^ | 0.80 (0.49 to 1.32) | Reference | 1.08 (0.63 to 1.84) |
| Never smoker |  |  |  |
| Events/Person-years | 138/3941 | 486/14849 | 106/3650 |
| AHR (95% CI) ^a^ | 1.10 (0.90 to 1.35) | Reference | 0.80 (0.65 to 1.00) |
| Alcohol intake status |  |  |  |
| Current drinker |  |  |  |
| Events/Person-years | 62/1499 | 179/4726 | 39/922 |
| AHR (95% CI) ^a^ | 1.03 (0.76 to 1.41) | Reference | 1.13 (0.79 to 1.60) |
| Former drinker |  |  |  |
| Events/Person-years | 5/233 | 20/769 | 7/168 |
| AHR (95% CI) ^a^ | 0.80 (0.25 to 2.57) | Reference | 1.50 (0.60 to 3.76) |
| Never drinker |  |  |  |
| Events/Person-years | 147/4052 | 512/14731 | 117/3724 |
| AHR (95% CI) ^a^ | 1.04 (0.85 to 1.26) | Reference | 0.83 (0.68 to 1.02) |
| Diabetes |  |  |  |
| YES |  |  |  |
| Events/Person-years | 42/601 | 129/2325 | 28/831 |
| AHR (95% CI) ^a^ | 1.24 (0.85 to 1.80) | Reference | 0.61 (0.40 to 0.93) |
| NO |  |  |  |
| Events/Person-years | 173/5189 | 583/17910 | 135/3983 |
| AHR (95% CI) ^a^ | 0.98 (0.82 to 1.18) | Reference | 1.02 (0.84 to 1.23) |
| Hypertension |  |  |  |
| YES |  |  |  |
| Events/Person-years | 98/1929 | 271/6154 | 69/1537 |
| AHR (95% CI) ^a^ | 1.21 (0.94 to 1.55) | Reference | 0.97 (0.74 to 1.26) |
| NO |  |  |  |
| Events/Person-years | 117/3862 | 441/14081 | 94/3277 |
| AHR (95% CI) ^a^ | 0.91 (0.73 to 1.13) | Reference | 0.88 (0.70 to 1.11) |
| Hyperlipidemia |  |  |  |
| YES |  |  |  |
| Events/Person-years | 109/2098 | 296/7013 | 72/1855 |
| AHR (95% CI) ^a^ | 1.26 (0.99 to 1.60) | Reference | 0.88 (0.68 to 1.14) |
| NO |  |  |  |
| Events/Person-years | 106/3693 | 416/13222 | 91/2960 |
| AHR (95% CI) ^a^ | 0.85 (0.68 to 1.07) | Reference | 0.95 (0.76 to 1.20) |

Abbreviations: CVD, cardiovascular disease; AHR, adjusted hazard ratio.

^a^ Hazard ratios were stratified for age at risk, gender, and adjusted for baseline heart rate, BMI, smoking status, alcohol intake status, education, physical activity, hypertension, hyperlipidemia, family history of CVD, diabetes, except for the stratified variable itself.

| **Supplementary Table 12.** Adjusted hazard ratios (HRs) for cardiovascular events by baseline heart rate and change of heart rate | | | | | | | | | | | |
| --- | --- | --- | --- | --- | --- | --- | --- | --- | --- | --- | --- |
| **Outcomes** | **Change of heart rate** | | | | | | | | | | |
|  | **Baseline heart rate <65 bpm** | | |  | **Baseline heart rate 65-80 bpm** | | |  | **Baseline heart rate >80 bpm** | | |
|  | **<-5 bpm** | **-5 to 15 bpm** | **>15 bpm** |  | **<-5 bpm** | **-5 to 15 bpm** | **>15 bpm** |  | **<-5 bpm** | **-5 to 15 bpm** | **>15 bpm** |
| CVD |  |  |  |  |  |  |  |  |  |  |  |
| Events/Person-years | 9/109 | 102/2620 | 30/1027 |  | 137/3911 | 540/15891 | 116/3537 |  | 69/1771 | 70/1724 | 17/249 |
| AHR (95% CI) ^a^ | 2.48  (1.27 to 4.82) | 1.08  (0.87 to 1.33) | 0.83  (0.57 to 1.20) |  | 0.98  (0.81 to 1.18) | Reference | 0.90  (0.74 to 1.11) |  | 1.07  (0.83 to 1.37) | 1.08  (0.84 to 1.39) | 1.67  (1.02 to 2.71) |
| CHD |  |  |  |  |  |  |  |  |  |  |  |
| Events/Person-years | 5/114 | 80/2662 | 25/1032 |  | 118/3945 | 444/16028 | 97/3565 |  | 56/1796 | 60/1742 | 13/250 |
| AHR (95% CI) ^a^ | 1.52  (0.62 to 3.68) | 1.02  (0.80 to 1.30) | 0.83  (0.56 to 1.25) |  | 1.03  (0.84 to 1.26) | Reference | 0.92  (0.74 to 1.15) |  | 1.06  (0.80 to 1.40) | 1.14  (0.87 to 1.49) | 1.59  (0.91 to 2.78) |
| ACS |  |  |  |  |  |  |  |  |  |  |  |
| Events/Person-years | 2/114 | 25/2662 | 8/1032 |  | 45/3945 | 133/16028 | 33/3565 |  | 21/1796 | 23/1742 | 2/250 |
| AHR (95% CI) ^a^ | 1.78  (0.43 to 7.30) | 1.07  (0.69 to 1.64) | 0.83  (0.41 to 1.71) |  | 1.28  (0.91 to 1.80) | Reference | 1.02  (0.69 to 1.49) |  | 1.28  (0.80 to 2.03) | 1.36  (0.87 to 2.13) | 0.78  (0.19 to 3.16) |
| Stroke |  |  |  |  |  |  |  |  |  |  |  |
| Events/Person-years | 4/119 | 22/2759 | 5/1080 |  | 19/4117 | 96/16711 | 19/3719 |  | 13/1867 | 10/1838 | 4/269 |
| AHR (95% CI) ^a^ | 6.17  (2.23 to 17.12) | 1.28  (0.80 to 2.04) | 0.84  (0.34 to 2.06) |  | 0.75  (0.45 to 1.22) | Reference | 0.85  (0.52 to 1.40) |  | 1.06  (0.59 to 1.89) | 0.82  (0.43 to 1.59) | 1.97  (0.72 to 5.41) |

Abbreviations: CVD, cardiovascular disease; CHD, coronary heart disease; ACS, acute coronary syndrome; AHR, adjusted hazard ratio.

^a^ Hazard ratios were stratified for age at risk, gender, and adjusted for BMI, smoking status, alcohol intake status, education, physical activity, hypertension, hyperlipidemia, family history of CVD, diabetes.

**Supplementary Figure 1.** Flow of participants in analyses of baseline heart rate and change of heart rate

(a) Flow of participants in analyses of baseline heart rate


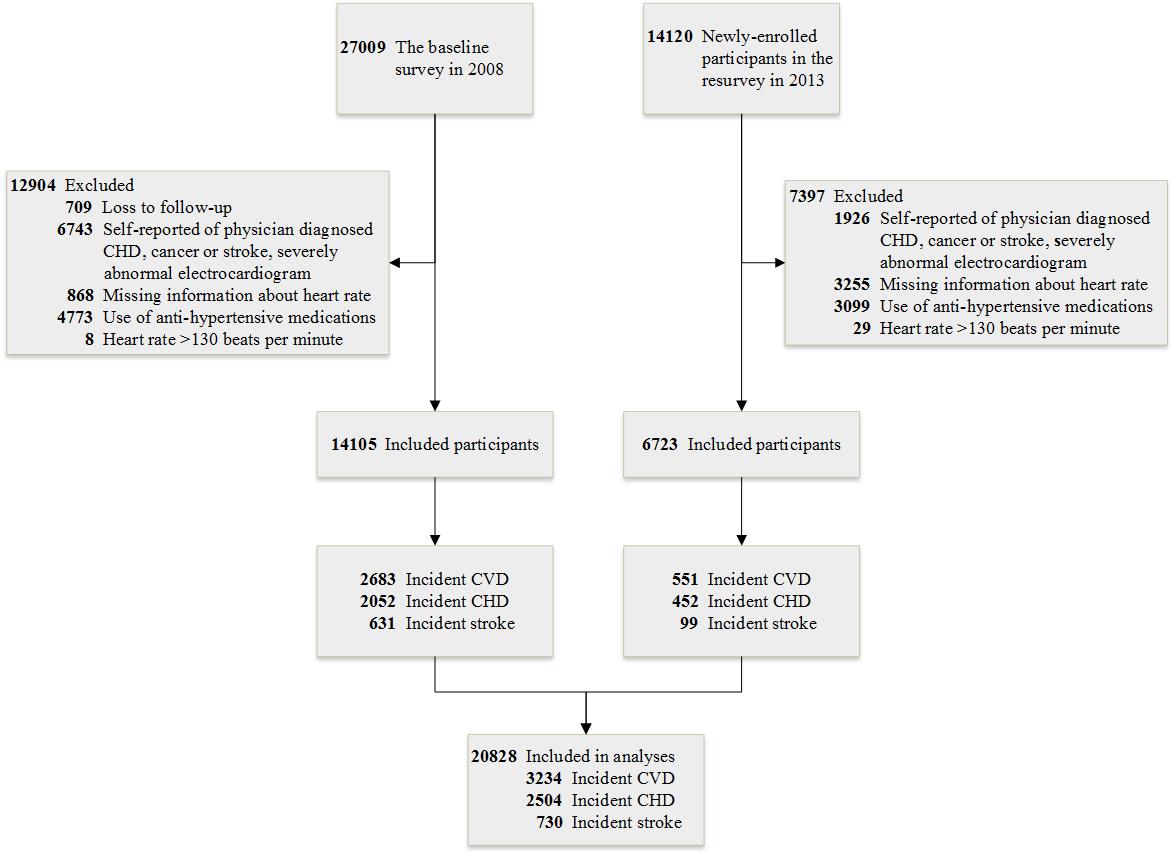


(b) Flow of participants in analyses of change of heart rate


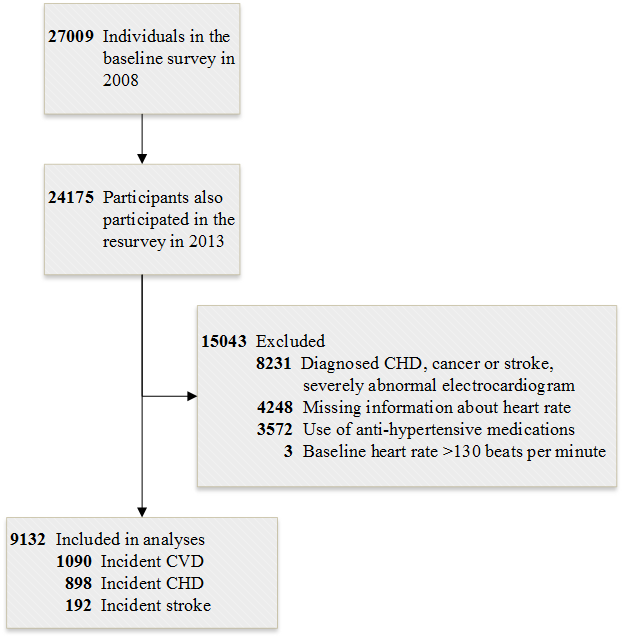


**Supplementary Figure 2.** Distribution of baseline heart rate and change of heart rate by gender

(a) Distribution of baseline heart rate


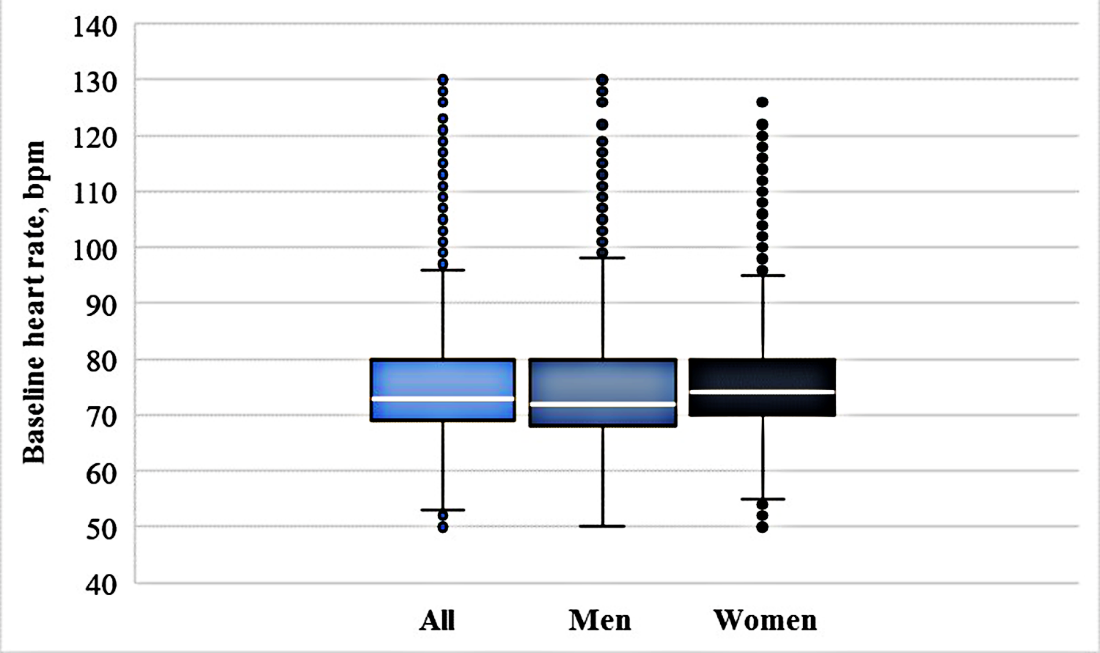


(b) Distribution of change of heart rate


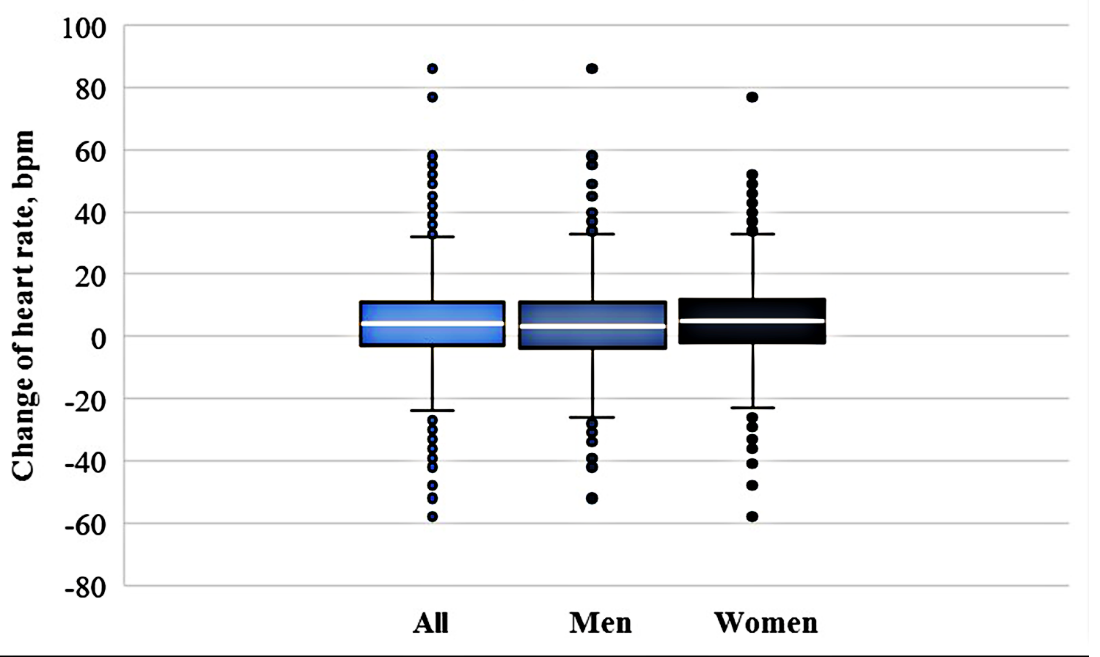

Supplement: Supplementary file 1 — SUPPLEMENTARY INFO [file 41598_2019_43045_MOESM1_ESM.docx]
